# Stem Cell Mobilization and Autologous Transplantation Outcomes After First-Line R-DA-EPOCH in High-Risk DLBCL and HGBCL: A Single-Center Retrospective Study

**DOI:** 10.3390/cancers18121874

**Published:** 2026-06-08

**Authors:** Daniela Taurino, Giulia Figini, Francesca Ricci, Chiara De Philippis, Barbara Sarina, Laura Giordano, Monica Balzarotti, Daniele Mannina, Arianna Giacomel, Ilenia De Bernardis, Armando Santoro, Stefania Bramanti

**Affiliations:** 1 Bone Marrow Unit, Humanitas Cancer Center, IRCCS Humanitas Research Hospital, via Manzoni 56, Rozzano, 20089 Milan, Italy; francesca.ricci@humanitas.it (F.R.); chiara.de_philippis@humanitas.it (C.D.P.); barbara.sarina@humanitas.it (B.S.); monica.balzarotti@humanitas.it (M.B.); daniele.mannina@humanitas.it (D.M.); arianna.giacomel@humanitas.it (A.G.); ilenia.debernardis@cancercenter.humanitas.it (I.D.B.); armando.santoro@humanitas.it (A.S.); stefania.bramanti@humanitas.it (S.B.); 2 Department of Biomedical Sciences, Humanitas University, via Rita Levi Montalcini 4, Pieve Emanuele, 20072 Milan, Italy; 3 Biostatistic Unit, Humanitas Cancer Center, IRCCS Humanitas Research Hospital, via Manzoni 56, Rozzano, 20089 Milan, Italy; laura.giordano@humanitas.it

**Keywords:** DLBCL, high-grade B-cell lymphoma, stem cell mobilization, autologous stem cell transplantation, R-DA-EPOCH

## Abstract

Background/Objectives: Patients with high-risk diffuse large B-cell lymphoma (DLBCL) and high-grade B-cell lymphoma (HGBCL) have suboptimal outcomes with standard therapies. Dose-adjusted R-EPOCH (R-DA-EPOCH) is often used in selected high-risk settings and may also serve as an effective chemo-mobilizing strategy for autologous stem cell transplantation (ASCT). We aimed to evaluate the impact of first-line R-DA-EPOCH on stem cell mobilization and transplantation outcomes. Methods: We retrospectively analyzed 41 consecutive adult patients with high-risk DLBCL/HGBCL treated with first-line R-DA-EPOCH between 2016 and 2021. Stem cell collection was planned after cycle 5. The primary endpoint was the rate of poor mobilizers, defined as patients who collected <2 × 10^6^ CD34^+^ cells/kg or required plerixafor (PLX) to achieve this threshold. Results: The rate of poor mobilizers was 31.7% (13/41), with 22% (9/41) of patients requiring PLX. Overall, 68% of patients achieved ≥2 × 10^6^ CD34^+^ cells/kg at first mobilization, while 88% ultimately reached the target after remobilization. Mobilization failure occurred in 12% of patients. A total of 59% of patients proceeded to ASCT. Engraftment was achieved in all transplanted patients, with no transplant-related mortality. Conclusions: First-line R-DA-EPOCH enables effective stem cell mobilization in most high-risk DLBCL and HGBCL patients, although a substantial proportion require PLX support. These findings suggest that R-DA-EPOCH is a feasible platform for early mobilization and ASCT in selected patients.

## Simple Summary

Patients with aggressive B-cell lymphomas and high-risk biological features often have suboptimal outcomes with standard treatments. Dose-adjusted R-EPOCH is frequently used in this setting and may also serve as a platform for stem cell mobilization. We analyzed whether first-line R-DA-EPOCH allows effective stem cell collection and safe autologous transplantation. Most patients achieved adequate stem cell yields; transplantation was feasible in over half of the cohort, and engraftment was predictable, with no transplant-related mortality.

## 1. Introduction

Diffuse large B-cell lymphoma (DLBCL) is the most common aggressive subtype of non-Hodgkin lymphoma in adults, accounting for approximately 30–40% of new lymphoma diagnoses worldwide. Despite major advances with immunochemotherapy regimens such as R-CHOP, approximately one-third of patients develop relapsed or refractory disease [1]. In this setting, salvage therapy followed by high-dose chemotherapy and autologous stem cell transplantation (ASCT) remains a cornerstone of treatment for eligible patients experiencing relapse more than 12 months after first-line therapy, a setting in which CAR-T cell therapy is not currently approved [2].

While R-CHOP remains the first-line standard in most settings, intensified regimens such as dose-adjusted R-EPOCH (R-DA-EPOCH) have been investigated in selected high-risk patients, such as “double expressor” (MYC and BCL2 co-overexpression) [3] and “double-hit” [4] or “triple-hit” [5] lymphomas (harboring MYC and BCL2 and/or BCL6 rearrangements), which represent biologically aggressive subgroups associated with poor outcomes following R-CHOP [6,7,8]. In this context, consolidation with high-dose chemotherapy followed by ASCT has been explored as a strategy to enhance long-term disease control in patients achieving remission after induction therapy.

Upfront ASCT has been explored as a consolidation strategy in selected cohorts of patients with biologically high-risk DLBCL treated with standard immunochemotherapy. However, available evidence remains heterogeneous, and this approach has not been uniformly incorporated into routine first-line management [9,10].

Successful ASCT depends on the effective mobilization and collection of hematopoietic stem and progenitor cells (HSPC), typically quantified by peripheral blood CD34^+^ cell counts [11,12]. Peripheral blood stem cells (PBSC) have largely replaced bone marrow as the preferred graft source, owing to faster hematologic recovery and lower infectious risk [13].

Mobilization is most commonly achieved using granulocyte colony-stimulating factor (G-CSF), alone or in combination with chemotherapy, to promote HSPC egress from the bone marrow niche [14]. However, up to 20% of patients fail to mobilize sufficient CD34^+^ cells, a scenario that complicates transplantation logistics and adversely impacts outcomes [15].

Plerixafor (PLX), a CXCR4 antagonist, has proven highly effective as an adjunct to G-CSF in poor mobilizers or those at high risk of mobilization failure, significantly increasing the likelihood of collecting adequate stem cell doses while reducing the number of apheresis sessions [16,17]. Risk factors for mobilization failure include older age, extensive prior chemotherapy, prior radiotherapy, bone marrow involvement, and low baseline peripheral blood CD34^+^ counts [18]. Early identification of at-risk patients and tailored mobilization strategies are therefore essential to ensure timely transplantation and favorable outcomes. In the prospective multicenter GOA study, mobilization parameters such as peripheral blood CD34^+^ counts >32 × 10^6^/L and first-apheresis CD34^+^ yields >2.75 × 10^6^/kg were associated with faster hematologic recovery and improved OS [19].

Despite advances in mobilization protocols, considerable variability in institutional practice persists, and the optimal mobilization regimen in DLBCL remains undefined. Furthermore, the role of ASCT itself is being re-evaluated in the era of CAR T-cell therapy and novel agents, requiring a reassessment of mobilization strategies within this evolving therapeutic landscape [20].

In the present study, we report the results of a single-center retrospective analysis to evaluate the impact of R-DA-EPOCH on HSPC collection and post-transplant engraftment in patients with DLBCL or high-grade B-cell lymphoma (HGBCL) who carry high-risk biological features and were treated with R-DA-EPOCH induction.

## 2. Materials and Methods

### 2.1. Setting and Design

This single-center retrospective observational study evaluated stem cell mobilization following EPOCH-based chemotherapy in patients with DLBCL or HGBCL harboring high-risk biological features. Eligible patients were required to have at least one of the following high-risk characteristics: “double expressor” [3], “triple expressor” [21], “double hit” [4], “triple hit” [5], TP53 mutation [22], 17p deletion [23], high Revised-International Prognostic Index (R-IPI) score [24], and intermediate–high National Comprehensive Cancer Network–International Prognostic Index (NCCN-IPI) score [25].

A total of 41 consecutive adult patients treated between May 2016 and September 2021 were included in the analysis. All patients received dose-adjusted R-EPOCH chemotherapy as first-line treatment and were subsequently evaluated for stem cell mobilization. Patients who achieved complete remission (CR) after induction were eligible for consolidation with ASCT.

The Institutional Review Board of the IRCCS Humanitas Research Hospital approved the study. Due to the retrospective observational design of the study, the requirement for study-specific informed consent for the collection and analysis of retrospective clinical data was waived in accordance with institutional policies and applicable regulations. All procedures were performed in accordance with the Ethical Standards of the Responsible Committee on Human Experimentation (institutional and national) and the Helsinki Declaration of 1975, as revised in 2008.

### 2.2. Patients

All patients had a histologically confirmed DLBCL or HGBCL diagnosis established according to the 2016 World Health Organization (WHO) classification of lymphoid neoplasms [26]. Additional inclusion criteria were signed informed consent for stem cell mobilization and harvesting, age ≥18 and <75 years, eligibility for ASCT, treatment with first-line DA-EPOCH, and complete response (CR) according to Lugano response criteria at the time of ASCT [27]. Exclusion criteria included patients who underwent stem cell mobilization after ≥2 lines of therapy, patients who were unable to understand and sign the informed consent, and patients with stable or progressive disease.

### 2.3. Stem Cell Mobilization and Collection

PBSC mobilization was performed using the R-DA-EPOCH regimen itself as chemo-mobilization. After completion of the fifth cycle of R-DA-EPOCH, patients underwent stem cell collection during hematologic recovery. Peripheral blood CD34^+^ cell counts were monitored from day +10 after chemotherapy, and leukapheresis was initiated when circulating CD34^+^ cell counts exceeded 10–20 × 10^6^/L.

Patients who failed to mobilize adequately after EPOCH-based chemo-mobilization received subsequent mobilization with granulocyte colony-stimulating factor (G-CSF, 10 µg/kg/day subcutaneously for 4–5 days). In cases of persistent mobilization failure despite G-CSF alone, a cyclophosphamide-based regimen (1.5 g/m^2^) was administered as salvage mobilization chemotherapy.

The goal of mobilization was to collect ≥2 × 10^6^ CD34^+^ cells/kg as the minimum target for ASCT, with ≥4 × 10^6^ CD34^+^ cells/kg as the optimal yield. Patients who failed to achieve sufficient mobilization with G-CSF ± chemotherapy were eligible for on-demand PLX (0.24 mg/kg) when peripheral blood CD34^+^ cell counts were between 5 and 19 cells/µL. Apheresis was performed using continuous-flow cell separators, and CD34^+^ cell enumeration was assessed by flow cytometry in accordance with ISHAGE guidelines [28].

### 2.4. Conditioning Regimen and Transplantation

**Figure 1:**
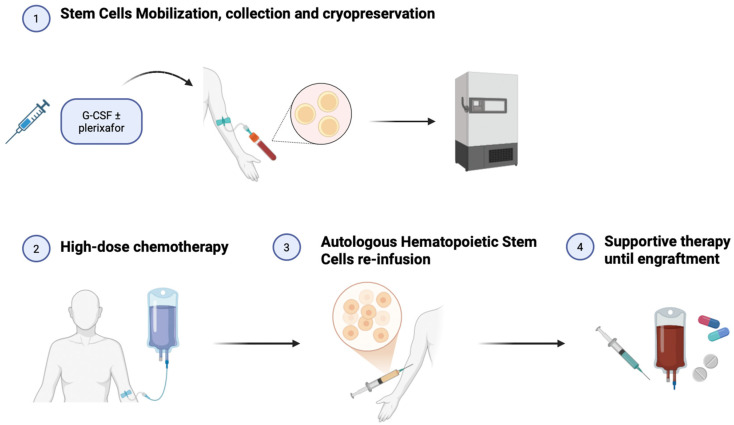
Schematic representation of the autologous stem cell transplantation (ASCT) process. (**1**) PBSC mobilization with chemotherapy and/or growth factors (G-CSF ± plerixafor), followed by leukapheresis and cryopreservation. (**2**) Delivery of high-dose chemotherapy. (**3**) Infusion of autologous hematopoietic stem cells. (**4**) Supportive care during the aplastic phase until hematologic engraftment.

High-dose chemotherapy was administered as conditioning prior to ASCT using FEAM (fotemustine, etoposide, cytarabine, melphalan) [29]. Cryopreserved PBSCs were reinfused on day 0, and G-CSF was reinitiated from day +5 only in 10patients who received <5 × 10^6^ CD34^+^ cells/kg, until neutrophil recovery (absolute neutrophil count > 0.5 × 10^9^/L for three consecutive days) [30,31]. Engraftment was defined as the first of three consecutive days with neutrophil or platelet recovery above thresholds of 0.5 × 10^9^/L and 20 × 10^9^/L, respectively, without transfusion support. Toxicities were graded according to the National Cancer Institute Common Terminology Criteria for Adverse Events (CTCAE), version 5.0. Mucositis, diarrhea, hepatic toxicity, and gastrointestinal toxicity were defined and graded according to these criteria. Viral reactivation was defined according to institutional monitoring protocols and standard clinical practice, including detection of viral DNAemia above clinically relevant thresholds (Figure 1).

### 2.5. Response Assessment and Endpoints

The study’s primary endpoint was the rate of poor mobilizers (PM), defined as patients who collected <2 × 10^6^ CD34^+^ cells/kg or required PLX to achieve ≥2 × 10^6^ CD34^+^ cells/kg.

The secondary endpoints included the rate of PLX use, engraftment rate after ASCT, rates of suboptimal (2–4 × 10^6^ CD34^+^ cells/kg) and optimal (>4 × 10^6^ CD34^+^ cells/kg) collections, the proportion of patients who did not undergo ASCT due to mobilization failure, the number of apheresis sessions required to obtain ≥2 × 10^6^ CD34^+^ cells/kg, the median CD34^+^ cell yield, the identification of factors associated with poor mobilization and PLX use, progression-free survival (PFS) and overall survival (OS).

### 2.6. Statistical Analysis

Continuous variables were summarized as median and range, or as mean and range, while categorical variables were reported as counts and percentages. Associations between clinical and biological variables and the risk of poor mobilization were examined using univariate logistic regression models, and results were reported as odds ratios (ORs) with 95% confidence intervals (CIs). PFS and OS were estimated using the Kaplan–Meier method. Survival curves were compared descriptively. Median follow-up was calculated using the reverse Kaplan–Meier method. A two-sided *p*-value < 0.05 was considered statistically significant. All statistical analyses were performed using SAS software, version 9.4 (SAS Institute, Cary, NC, USA).

## 3. Results

A total of 41 patients who underwent hematopoietic stem cell mobilization between September 2016 and March 2022 were included in this analysis. Baseline demographic and clinical characteristics are reported in Table 1.

The overall rate of poor mobilizers was 31.7% (13/41), and 22% (9/41) of patients required PLX at any point during mobilization. A total of 68% (28/41) of patients successfully collected at least 2 × 10^6^ CD34^+^ cells/kg during the first mobilization attempt. The proportion of patients achieving an optimal collection, with or without PLX, was 73% (30/41), whereas 15% (6/41) achieved a suboptimal collection. Mobilization failure precluded ASCT in 12% of patients (5/41).

**Figure 2:**
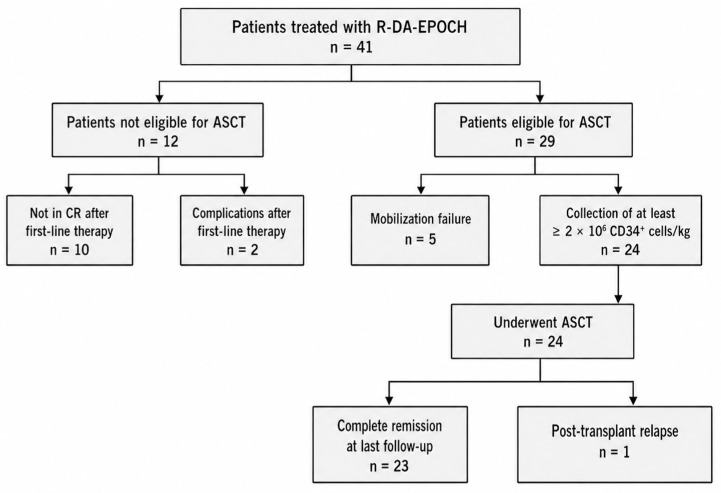
Flow diagram of patient selection, mobilization, and ASCT outcomes.

The median CD34^+^ cell yield was 5.7 × 10^6^/kg (range 0.6–14 × 10^6^/kg). The median number of apheresis sessions required was 2 (range 1–3). A total of 11 patients failed to reach the threshold of 2 × 10^6^ CD34^+^ cells/kg. Among them, six patients were successfully remobilized with G-CSF alone, cyclophosphamide plus G-CSF, or on-demand PLX. Overall, 88% (36/41) of the cohort ultimately achieved a collection of at least 2 × 10^6^ CD34^+^ cells/kg, and 59% (24/41) of patients proceeded to ASCT. Mobilization and collection outcomes are summarized in Table 2. Overall, 10 patients did not proceed to ASCT because they failed to achieve CR after chemotherapy; 1 patient was excluded due to persistent cytopenias following chemotherapy; and 1 patient did not undergo ASCT due to a prolonged SARS-CoV-2 infection after completion of the last cycle of R-DA-EPOCH (Figure 2).

Engraftment outcomes were evaluated in all transplanted patients. The median CD34^+^ cell dose infused was 5.9 × 10^6^/kg. Hematopoietic recovery was achieved in all transplanted patients. The median time to neutrophil engraftment was 15 days (range 11–33), while the median time to platelet recovery was 17 days (range 9–120).

Overall, the transplant procedure was well tolerated, and no transplant-related mortality was observed. Infectious complications represented the most common toxicities: 75% (18/24) of patients experienced a fever of unknown origin (FUO), 21% (5/24) developed sepsis, and 12% (3/24) developed pneumonia. Viral reactivations occurred in 29% (7/24) of patients, predominantly involving HHV-6, CMV, and Herpes Zoster. All patients developed mucositis, with a maximum severity of grade 3. Diarrhea occurred in 71% of patients (17/24), although grade 3 diarrhea was observed in only 17% (4/24). Hepatic toxicity of grade 3 was documented in 12% (3/24) of cases. Two patients experienced cardiac adverse events (one episode of atrial fibrillation and one of acute circulatory decompensation), and one patient developed neutropenic enterocolitis complicated by intestinal obstruction. Most adverse events resolved within three months following transplantation.

**Figure 3:**
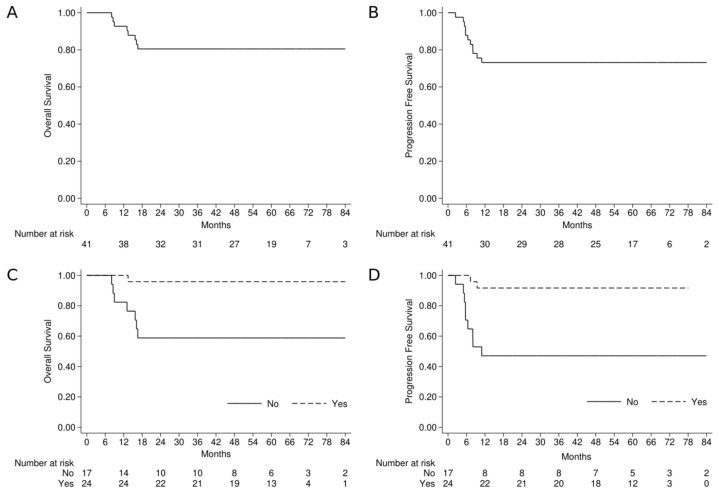
Overall survival (OS) and progression-free survival (PFS). OS (**A**) and PFS (**B**) in the intention-to-treat population; OS (**C**) and PFS (**D**) according to autologous stem cell transplantation (yes vs. no).

With a median follow-up of 64 months, 5-year PFS and OS among transplanted patients were 92% and 96%, respectively. Among non-transplant patients, PFS and OS were 47% and 59%. In the intention-to-treat (ITT) population, PFS was 73%, and OS was 81% (Figure 3).

A univariate analysis was performed to evaluate the association between poor mobilization and consequent PLX use with selected clinical variables. The presence of B symptoms at diagnosis (OR 0.83, *p* = 0.7905), bulky disease (OR 1.13, *p* = 0.8651), the presence of a TP53 mutation (OR 1.19, *p* = 0.8172) or chromosome 17 deletion (OR 2.67, *p* = 0.2786), non-GC histology (OR 1.8, *p* = 0.40), an R-IPI ≥4 (OR 1.02, *p* = 0.9585), an NCCN-IPI ≥4 (OR 1.18, *p* = 0.5329), disease stage ≥3 (OR 0.75, *p* = 0.6985), extranodal involvement (OR 0.38, *p* = 0.2248), bone marrow involvement (OR 0.5, *p* = 0.436), and CNS-IPI (OR 0.59, *p* = 0.463) were not significantly associated with the risk of poor mobilization. Conversely, although not statistically significant, increasing age (OR 1.1, *p* = 0.096) showed a trend toward an increased risk of poor mobilization and consequent PLX use.

## 4. Discussion

In this single-center retrospective study, we evaluated the feasibility and effectiveness of hematopoietic stem cell mobilization following first-line R-DA-EPOCH in patients with DLBCL and HGBCL harboring high-risk biological features. Our results indicate that R-DA-EPOCH supports effective stem cell mobilization and collection in the majority of patients, with an overall mobilization failure rate of 12% and reliable engraftment in all transplanted individuals. An important finding emerging from our analysis is that the main barrier to proceeding with ASCT was not stem cell mobilization failure but rather inadequate disease response after induction therapy. This finding suggests that, in this biologically adverse population, disease biology appears to remain the primary determinant of transplant feasibility, whereas hematopoietic reserve appears largely preserved when mobilization is planned early during first-line treatment. Rescue strategies, including G-CSF alone, chemotherapy-based mobilization, and on-demand PLX, further improved the likelihood of achieving an adequate CD34^+^ cell yield.

When compared with other chemo-mobilizing regimens commonly used in lymphoma, such as ICE, DHAP, or high-dose cyclophosphamide, R-DA-EPOCH appears to provide comparable or superior mobilization, with the additional advantage of serving as an effective induction regimen for aggressive B-cell lymphomas. Regimens such as ICE or DHAP are widely used for stem cell mobilization; however, they are traditionally administered as second-line salvage therapies and therefore typically involve patients who have already shown a suboptimal response to first-line treatment [32,33]. In contrast, R-DA-EPOCH allows stem cell collection during first-line remission, potentially improving mobilization efficiency and clinical outcomes [34,35]. Moreover, historical data suggest that mobilization after multi-agent salvage chemotherapy may be impaired in heavily pretreated patients [36,37], whereas mobilization after first-line intensive regimens may preserve bone marrow reserve more effectively. In this context, EPOCH-based chemo-mobilization represents a biologically and logistically rational approach, especially for high-risk subsets.

The rate of poor mobilizers observed in our study was higher than the proportion of patients who ultimately failed to proceed to ASCT, reflecting our pragmatic definition of poor mobilization, which also included patients requiring plerixafor support. While this definition differs partially from consensus-based criteria, it mirrors real-world clinical practice, where early PLX use is increasingly adopted to optimize collection efficiency and avoid mobilization delays. Importantly, despite the relatively high rate of PLX utilization, the majority of patients achieved optimal or suboptimal collections sufficient for transplantation.

Despite the relatively small sample size, our findings also support the notion that ASCT remains a safe and well-tolerated consolidative strategy, with acceptable toxicity and encouraging long-term outcomes in appropriately selected patients. Neutrophil and platelet engraftment were achieved within expected timeframes, and no unexpected transplantation-related complications were observed.

However, these findings should be interpreted in light of several limitations. In particular, information bias cannot be excluded, as molecular and cytogenetic characterization evolved over the study period, and high-risk features were not uniformly assessed in earlier years, potentially leading to an underestimation of biological risk. Moreover, the relatively favorable mobilization and transplant outcomes observed in our study may in part reflect the selection of patients with adequate performance status and treatment response. Finally, residual confounding related to disease biology, treatment exposure, and supportive care may have impacted the results, particularly given the limited sample size and the absence of multivariable analysis.

The presence of bulky disease, TP53 mutation, or chromosome 17 deletion, non-GC histology, and high-risk prognostic scores could plausibly be associated with an increased likelihood of poor mobilization, given their established relationship with aggressive disease biology and reduced hematopoietic reserve. In our cohort, however, none of these variables, including age, demonstrated a statistically significant association with mobilization failure, likely due to the limited sample size. Nevertheless, these findings should be interpreted within a broader biological framework. High-risk genomic features may affect not only disease aggressiveness but also treatment exposure and bone marrow reserve, thereby indirectly influencing stem cell mobilization and transplant outcomes [38]. An integrated approach combining molecular, clinical, and treatment-related factors could improve risk stratification and support personalized mobilization strategies, including the early use of PLX. Future studies incorporating multi-omics data are warranted.

Although the therapeutic landscape has evolved significantly with the advent of CAR T-cell therapies, bispecific antibodies [39,40,41], and other regimens [42,43], it is important to recognize that a non-negligible proportion of patients remain ineligible for these novel treatments. Contraindications such as central nervous system (CNS) involvement, pre-existing neurological comorbidities (e.g., epilepsy), or autoimmune diseases requiring specific immunosuppression may limit the feasibility or safety of T-cell–redirecting therapies. For these patients, particularly those with biologically high-risk disease as represented in our cohort, frontline consolidation with ASCT may still offer a meaningful therapeutic advantage by providing durable disease control in a setting where alternative options are limited or contraindicated.

Taken together, our results suggest that R-DA-EPOCH not only provides effective cytoreduction in high-risk DLBCL and HGBCL but also supports efficient hematopoietic stem cell mobilization, enabling timely autologous transplantation in eligible patients. In an era of rapidly expanding therapeutic innovations, ASCT remains a valuable and potentially underutilized strategy for selected high-risk patients, especially those who cannot access or safely receive novel immunotherapies. However, these findings should be interpreted with caution, given the retrospective design, limited sample size, and absence of a comparator cohort. Larger prospective multicenter studies integrating clinical, genomic, and transcriptomic data are warranted to validate these findings and to refine the positioning of ASCT within contemporary risk-adapted treatment strategies for aggressive B-cell lymphomas.

## 5. Conclusions

In selected patients with high-risk DLBCL and HGBCL, first-line dose-adjusted R-EPOCH enables effective hematopoietic stem cell mobilization and supports safe autologous stem cell transplantation with predictable engraftment and acceptable toxicity. Although disease biology appears to be a major determinant of transplant eligibility, early mobilization following R-DA-EPOCH may help preserve hematopoietic reserve and allows successful stem cell collection in most patients.

In the current era of rapidly expanding therapeutic options, ASCT continues to represent a relevant consolidative option for carefully selected patients who achieve an adequate response and are not candidates for novel immunotherapies. Prospective multicenter studies are warranted to better define the optimal integration of R-DA-EPOCH, stem cell mobilization strategies, and ASCT within contemporary risk-adapted treatment algorithms for aggressive B-cell lymphomas.

## Figures and Tables

**Table 1:** Baseline demographic and clinical characteristics.

Characteristics	Value
Age at diagnosis median (range)	58 (22–69)
Gender N (%)	
Male	22 (54%)
Female	19 (46%)
Histopathologic category N (%)	
DLBCL, NOS	30 (73%)
HGBCL	11 (27%)
Ann Arbor stage at diagnosis N (%)	
1–2	11 (27%)
3–4	30 (73%)
B symptoms at diagnosis N (%)	17 (41%)
Bulky disease N (%)	15 (37%)
Bone marrow infiltration N (%)	10 (24%)
Extranodal involvement N (%)	33 (80%)
Molecular subtypes N (%)	
GCB subtype	24 (58%)
Non-GCB subtype	16 (39%)
Unknown	1 (3%)
R-IPI risk group N (%)	
Very good (0)	1 (2%)
Good (1–2)	15 (37%)
Poor (3–5)	25 (61%)
NCCN-IPI risk group N (%)	
Low (0–1)	1 (2%)
Low–intermediate (2–3)	12 (29%)
High–intermediate (4–5)	22 (54%)
High (6–8)	6 (15%)
CNS-IPI risk group N (%)	
Low (0–1)	6 (15%)
Intermediate (2–3)	19 (46%)
High (4–6)	16 (39%)
MYC/BCL2/BCL6 expression status N (%)	
Double-expressor	8 (20%)
Triple-expressor	23 (56%)
MYC/BCL2/BCL6 rearrangement status N (%)	
Double-hit	7 (17%)
Triple-hit	1 (2%)
TP53 mutation N (%)	
Mutated	12 (29%)
Unmutated	27 (66%)
Unknown	2 (5%)
del (17p) N (%)	
Present	6 (15%)
Absent	33 (80%)
Unknown	2 (5%)
Disease status at EOT N (%)	
CR	31 (75%)
PR	2 (5%)
SD	2 (5%)
PD	6 (15%)
CNS prophylaxis N (%)	
HD-MTX	34 (83%)
IT	6 (15%)
None	1 (2%)
Radiotherapy N (%)	
Yes	3 (7%)
No	30 (73%)
Post-transplant	8 (20%)

**Table 2:** Mobilization and collection outcomes.

Parameter	Value
Number of apheresis sessions, median (range)	2 (1–3)
Total CD34^+^ cells collected, median (range), ×10^6^/kg	5.7 (0.6–14.0)
Optimal collection (≥4 × 10^6^ CD34^+^ cells/kg), N (%)	30 (73%)
Suboptimal collection (≥2 to <4 × 10^6^ CD34^+^ cells/kg), N (%)	6 (15%)
Mobilization failure (<2 × 10^6^ CD34^+^ cells/kg), N (%)	5 (12%)
Remobilization required, N (%)	13 (32%)
Patients required PLX at any time, N (%)	9 (22%)

## Data Availability

Data supporting the findings of this study are available from the corresponding authors upon reasonable request.
